# The dark side of Gen-AI-assisted clinical work collaboration: work alienation, trait emotional intelligence, and instigated interprofessional incivility

**DOI:** 10.3389/fpsyg.2026.1856755

**Published:** 2026-08-20

**Authors:** Zhanglan You, Pingping You, Isaac Wasonga Owino

**Affiliations:** 1 Business School, Fujian Business University, Fuzhou, China; 2 Department of Neurology, Zhongshan Hospital Xiamen University, Xiamen, China; 3 School of Business, University of Nairobi, Nairobi, Kenya

**Keywords:** Gen-AI-assisted clinical work collaboration, hospitals, instigated interprofessional incivility, physicians and nurses, trait emotional intelligence, work alienation

## Abstract

Generative artificial intelligence (Gen-AI) is becoming increasingly embedded in clinical work, yet its darker interpersonal and psychological consequences in hospital settings remain underexamined. Drawing on human–AI collaboration research and the strain process within job demands–resources (JD-R) theory, this study investigates whether Gen-AI-assisted clinical work collaboration increases instigated interprofessional incivility among physicians and nurses, whether work alienation mediates this relationship, and whether trait emotional intelligence (trait EI) conditions the alienation–incivility link. A three-wave time-lagged survey design was employed among physicians and nurses at Xiamen University Affiliated Zhongshan Hospital in China. After screening and cross-wave matching, 468 valid matched cases were retained and analyzed using SPSS 27.0, AMOS 24.0, and PROCESS 4.0. Results revealed that Gen-AI-assisted clinical work collaboration was positively associated with instigated interprofessional incivility and work alienation, and work alienation was positively associated with instigated interprofessional incivility. Work alienation partially mediated the relationship between Gen-AI-assisted clinical work collaboration and instigated interprofessional incivility. In addition, the interaction effects of self-emotion appraisal, others’ emotion appraisal, use of emotion, regulation of emotion, and overall trait EI on the relationship between work alienation and instigated interprofessional incivility were all significant. However, these effects emerged in the opposite direction from that hypothesized: higher levels of trait EI strengthened rather than weakened the positive relationship between work alienation and instigated interprofessional incivility. This study extends research on the dark side of Gen-AI-assisted work by showing that, in hospital settings, Gen-AI-assisted clinical work collaboration may increase instigated interprofessional incivility both directly and indirectly through work alienation. The findings also suggest that the role of trait EI in technologically mediated healthcare work is more context-dependent and ambivalent than a simple protective-resource interpretation would imply.

## 1 Introduction

Physicians and nurses work in healthcare environments characterized by high pressure, intense emotional labor, and persistent interprofessional coordination demands (World Health Organization, 2025). Their work requires not only technical competence but also sustained emotion regulation, rapid communication, and constant collaboration across professional boundaries (Powell et al., 2024). Emotional labor is a routine feature of healthcare work because clinicians must manage their emotions while caring for patients, interacting with families, and coordinating with colleagues under conditions of uncertainty and time pressure (Yeh et al., 2020). Related evidence further shows that workload, emotional demands, and organizational demands are associated with emotional exhaustion, depersonalization, and reduced work engagement among nurses (Montgomery et al., 2015). At the team level, effective interprofessional collaboration is critical for professional practice and healthcare outcomes, whereas poor collaboration can undermine service delivery and patient care (Reeves et al., 2017). Interprofessional incivility is therefore not a peripheral relational issue in hospitals. Rather, it has been linked to stress, compromised patient safety, and poorer quality of care among physicians and nurses (Lewis, 2023). This demanding and highly interdependent work context provides an important foundation for understanding how new digital technologies may reshape both clinicians’ work experience and their conduct toward colleagues (Chen and Kong, 2026).

Against this backdrop, generative artificial intelligence (Gen-AI) is becoming an increasingly visible part of healthcare work. Unlike broader forms of artificial intelligence that mainly classify, predict, or optimize, Gen-AI can generate new content such as summaries, draft notes, synthesized responses, and decision-support outputs. International guidance and emerging empirical studies suggest that Gen-AI is especially relevant to healthcare knowledge work, including documentation, information synthesis, and decision preparation, although its usefulness varies across tasks and workflows (Kharko et al., 2025; Ruan et al., 2025; World Health Organization, 2025). Emerging organizational research increasingly portrays Gen-AI collaboration as a double-edged phenomenon in healthcare. When generative systems become involved in drafting, summarizing, organizing, and suggesting, physicians and nurses may gain efficiency while simultaneously feeling less central to the cognitive and expressive core of their professional work. This possibility is especially important in hospitals, where technology-mediated work remains tightly coupled with professional accountability and relational conduct (Hermann et al., 2025). Although recent research has begun to identify the dark side of Gen-AI collaboration in workplaces, relatively little is known about how Gen-AI-assisted collaboration in hospitals may spill over into physician–nurse relations and shape enacted interprofessional conduct (Hai et al., 2025).

In the present study, Gen-AI-assisted clinical work collaboration refers to the extent to which generative AI systems are involved in and shape clinicians’ routine clinical and nursing work processes by contributing to documentation, information synthesis, communication drafting, decision preparation, and problem-solving activities. The construct therefore captures a broader pattern of technology-assisted collaboration within clinical work rather than a single task-specific function. In this sense, AI-supported problem-solving is treated as one important manifestation of Gen-AI-assisted clinical work collaboration, but not as its sole defining content. This conceptualization aligns with Anthony et al.’s (2023) system-level perspective on human–AI collaboration, which positions AI as a counterpart within work systems rather than a passive tool, thereby reshaping how tasks are divided, executed, and evaluated. It also resonates with Bankins et al.’s (2024) multilevel framework, which locates human–AI interaction along a substitution–augmentation–collaboration continuum and highlights collaboration as a distinct mode in which AI actively co-produces work outputs alongside human professionals. Furthermore, this conceptualization is consistent with the operationalization developed by Hai et al. (2025), who measured employee-GenAI collaboration through the perceived depth of AI involvement in routine work processes, and with Hermann et al.'s (2025) review of GenAI and the psychology of work, which emphasizes that generative systems alter not only task execution but also workers’ cognitive and relational engagement with their work.

Importantly, Gen-AI-assisted clinical work collaboration is not theoretically unidimensional within the job demands–resources (JD-R) framework. In hospital work, some aspects of such collaboration may function as resource-like conditions by supporting documentation efficiency, facilitating information synthesis, accelerating decision preparation, and reducing repetitive cognitive labor (Ruan et al., 2025; World Health Organization, 2025). At the same time, other aspects may function as demand-like conditions by increasing output verification burden, hallucination monitoring, accountability ambiguity, and coordination complexity (Hermann et al., 2025). The need to verify AI-generated clinical outputs for accuracy and reconcile machine-generated suggestions with professional standards introduces substantial cognitive vigilance and emotional tension into clinicians’ already demanding workflows (Goh et al., 2025). This distinction is important because JD-R theory is inherently dual-process: some work conditions facilitate functioning and goal attainment through the motivational process, whereas others consume cognitive, emotional, and relational resources through the health impairment process (Bakker and Demerouti, 2017). The present study does not deny the potential resource side of Gen-AI-assisted collaboration, which may enhance work engagement and performance under certain conditions (Bankins et al., 2024). Rather, it focuses specifically on its underexamined demand-inducing and strain-generating side in hospital settings, where the relational and psychological costs of technology-assisted collaboration may be especially consequential (Hai et al., 2025). This analytical focus is justified because, although the resource benefits of clinical AI are increasingly well-documented (Kharko et al., 2025; Ruan et al., 2025), its psychological costs in high-stakes healthcare environments remain substantially underexamined.

Understanding these demand-inducing and relational consequences carries both theoretical and practical significance. Theoretically, the integration of human–AI collaboration research with the strain process within JD-R theory offers a framework for explaining how technologically mediated work conditions in high-stakes professional environments may generate psychological costs that extend beyond task performance to shape interpersonal conduct (Bakker et al., 2023; Schaufeli and Taris, 2014). Practically, interprofessional incivility has been linked to compromised patient safety, impaired teamwork, and reduced quality of care (Freedman et al., 2025; Lewis, 2023), making it critical to understand whether and how Gen-AI-assisted collaboration may contribute to such relational deterioration as hospitals worldwide accelerate AI adoption (Hermann et al., 2025).

Accordingly, the present study develops and tests a model in which Gen-AI-assisted clinical work collaboration is positively associated with instigated interprofessional incivility both directly and indirectly through work alienation, while trait emotional intelligence and its four dimensions condition the relationship between work alienation and instigated interprofessional incivility. By focusing on physicians and nurses in a Chinese tertiary hospital, this study makes three contributions. First, it extends dark-side research on Gen-AI collaboration into healthcare and does so in a way that is grounded in contextualizing evidence about concrete Chinese hospital application scenarios. Second, it integrates human–AI collaboration research with the strain process within JD-R theory to explain why Gen-AI-assisted clinical work collaboration may foster work alienation and why alienation may spill over into instigated interprofessional incivility. Third, it moves beyond a global treatment of emotional intelligence by specifying how self-emotion appraisal, others’ emotion appraisal, use of emotion, and regulation of emotion may shape the relational risks of technologically mediated clinical work.

## 2 Literature review and hypothesis development

### 2.1 Gen-AI-assisted clinical work collaboration and instigated interprofessional incivility

Human–AI collaboration research suggests that contemporary AI systems should not be understood merely as passive tools that execute predefined commands (Chen and Kong, 2026). As AI increasingly participates in information generation, recommendation, and task execution, it functions as a consequential collaborator that reshapes how work is divided, enacted, and evaluated (Anthony et al., 2023). This perspective is especially relevant to Gen-AI, whose capacity to draft, summarize, synthesize, and suggest not only accelerates task completion but also changes workers’ relationship with the task itself. Employees may remain formally responsible for outcomes while becoming less directly involved in the cognitive and expressive processes through which those outcomes are produced. The automation–augmentation paradox further highlights this tension: AI may simultaneously enhance performance and reduce workers’ sense of ownership, agency, and responsibility over important aspects of their work (Raisch and Krakowski, 2021). In healthcare, these implications are especially pronounced because clinicians’ work is not exhausted by task completion alone (Ruan et al., 2025). Accordingly, when Gen-AI becomes involved in drafting clinical texts, organizing information, summarizing records, or preparing decision-support content, it may improve efficiency while also weakening clinicians’ sense of control, authorship, and meaningful engagement (Chen and Kong, 2026).

The strain process within JD-R theory complements this perspective by explaining why the demand-inducing aspects of Gen-AI-assisted clinical work collaboration may become psychologically straining (Bakker et al., 2023). JD-R theory posits that job demands require sustained physical, cognitive, or emotional effort and, when not adequately balanced by resources, can trigger strain processes and undermine wellbeing and functioning (Bakker and Demerouti, 2017; Bakker et al., 2023). In the context of Gen-AI-assisted clinical work collaboration, clinicians are not simply relieved of workload. Instead, new demands often emerge alongside the promised efficiencies. Physicians and nurses must verify AI-generated outputs, monitor hallucinations or inaccuracies, reconcile machine-generated suggestions with clinical standards and professional norms, and manage uncertainty regarding responsibility under conditions of incomplete algorithmic transparency (Anthony et al., 2023). These tasks introduce additional cognitive vigilance, emotional tension, and role ambiguity into already demanding hospital environments characterized by time pressure, interdependence, and high accountability (Bobak et al., 2021). Thus, the present study focuses on how the demand-inducing side of Gen-AI-assisted collaboration may consume psychological resources and foster work alienation, rather than attempting to empirically test the full dual-path JD-R model (Bakker et al., 2023; Schaufeli and Taris, 2014).

A key outcome examined in this study is instigated interprofessional incivility. Incivility has been classically defined as low-intensity deviant behavior with ambiguous intent to harm that violates workplace norms of mutual respect (Andersson and Pearson, 1999). Subsequent research further distinguished instigated incivility from merely experienced incivility by focusing on discourteous behavior enacted by the focal actor rather than only perceived from others (Blau and Andersson, 2005). In healthcare, this distinction is especially important because interprofessional work depends on timely, clear, and respectful exchanges among physicians, nurses, and other clinicians. A recent systematic review shows that interprofessional incivility is common in medical settings and is associated with stress, impaired teamwork, compromised patient safety, and poorer quality of care (Lewis, 2023; Freedman et al., 2025). Thus, in the present study, instigated interprofessional incivility refers specifically to low-intensity discourteous conduct enacted by physicians or nurses toward colleagues from another professional group during collaborative hospital work.

Gen-AI-assisted clinical work collaboration may directly heighten such enacted discourtesy because it can disrupt interaction patterns in addition to altering employees’ relationship with their work. Emerging research suggests that Gen-AI is not socially neutral; it can reshape discussion dynamics, coordination patterns, and influence processes within teams. For example, Gen-AI participation has been shown to alter group discussion and coordination by changing how collaborators exchange information, interpret contributions, and manage consensus in real time (Han et al., 2024; Wiss et al., 2025). In hospital work, where collaboration relies heavily on timely, clear, and respectful communication, such changes may directly strain interprofessional interaction when Gen-AI becomes involved in documentation, information synthesis, and decision preparation (Anthony et al., 2023). This direct relational disruption logic is consistent with human–AI collaboration research and the strain process within JD-R theory. When AI participates in task execution, it changes task ownership, participation, and responsibility experience, thereby reshaping how employees engage not only with the task but also with coworkers involved in the same work process (Raisch and Krakowski, 2021). At the same time, the associated monitoring, verification, and accountability demands may spill over directly into interactional conduct, appearing as impatience, curt replies, dismissiveness, or other forms of low-intensity discourtesy toward colleagues, even before a more enduring psychological state such as work alienation is fully formed (Bakker et al., 2023). This argument is also consistent with emerging research showing that incivility in human–AI contexts is morally salient and socially evaluated, suggesting that interactional norms remain consequential even when AI enters the exchange (Arundel and Young, 2023). Therefore, the present study proposes the following hypothesis:

*H1*: Gen-AI-assisted clinical work collaboration is positively associated with instigated interprofessional incivility.

### 2.2 Gen-AI-assisted clinical work collaboration and work alienation

The present study further argues that work alienation provides an important psychological mechanism linking Gen-AI-assisted clinical work collaboration to instigated interprofessional incivility. Work alienation refers to a psychological state in which employees experience their work as detached, externally shaped, and insufficiently meaningful or self-expressive (Nair and Vohra, 2010; Shantz et al., 2014; Hai et al., 2025). It is not equivalent to temporary dissatisfaction or simple dislike of a technology. Rather, it captures a deeper sense of estrangement from one’s work process, work role, and work outputs. Prior research has shown that work alienation is associated with reduced job satisfaction, weaker organizational attachment, emotional exhaustion, and other maladaptive outcomes, suggesting that it is a consequential form of work-related strain rather than a peripheral attitude (Chiaburu et al., 2014; Hirschfeld and Feild, 2000; Shantz et al., 2014). Alienation tends to emerge when workers experience diminished agency, reduced meaningful participation, and weaker identification with what they do.

These conditions are likely to become more salient under Gen-AI-assisted clinical work collaboration. Human–AI collaboration research suggests that when AI systems participate directly in drafting, summarizing, synthesizing, and suggesting, they increasingly become involved in the production of work itself (Anthony et al., 2023). For physicians and nurses, this means that important components of clinical and nursing work—such as documentation, information organization, preparatory reasoning, and communication drafting—may be co-produced with a generative system rather than fully generated through clinicians’ own cognitive and expressive labor (Ruan et al., 2025). Although clinicians retain formal responsibility for the final output, they may no longer experience the work process as fully authored or controlled by themselves. In this sense, Gen-AI-assisted clinical work collaboration may weaken felt ownership over tasks and reduce the extent to which work is experienced as directly self-expressive (Chen and Kong, 2026). From a JD-R perspective, Gen-AI-assisted clinical work collaboration also generates new cognitive, emotional, and role-related demands, because clinicians must verify AI-generated outputs, detect hallucinations, assess alignment with professional standards, and manage responsibility under conditions of incomplete algorithmic transparency (Bakker et al., 2023; Hai et al., 2025). Accordingly, Gen-AI-assisted clinical work collaboration is likely to heighten work alienation among physicians and nurses. This reasoning leads to the following hypothesis:

*H2*: Gen-AI-assisted clinical work collaboration is positively associated with work alienation.

### 2.3 Work alienation as a mediator

Work alienation should, in turn, be positively associated with instigated interprofessional incivility. Importantly, work alienation is not merely a negative attitude toward work; it also carries behavioral implications because it shapes how employees relate not only to their work itself but also to other people in the workplace. When employees feel alienated from their work, they are less likely to invest in work as meaningful, self-expressive, or normatively worthy of effort (Chiaburu et al., 2014; Shantz et al., 2014). As a result, alienated workers are less willing to devote discretionary patience, respect, and cooperation in everyday interactions, particularly under demanding conditions. In this sense, work alienation should be understood not only as a state of disconnection from work but also as a condition that can undermine the quality of employees’ interpersonal conduct in organizational life. This behavioral consequence is especially relevant in hospitals, where daily work is characterized by time pressure, frequent coordination, and high interdependence. Maintaining civility in interprofessional work often requires active self-regulation, perspective-taking, emotional control, and continued commitment to collaborative coordination under pressure (Lewis, 2023; Reeves et al., 2017). Work alienation undermines precisely these forms of discretionary relational investment. Therefore, the present study proposes the following hypotheses:

*H3*: Work alienation is positively associated with instigated interprofessional incivility.

Accordingly, the present study expects that work alienation will mediate the relationship between Gen-AI-assisted clinical work collaboration and instigated interprofessional incivility. Consistent with the strain process within JD-R theory, the demand-inducing side of Gen-AI-assisted collaboration may weaken clinicians’ sense of control, authorship, and meaningful engagement while simultaneously increasing monitoring, verification, and accountability demands, thereby heightening work alienation (Raisch and Krakowski, 2021). In turn, work alienation may erode clinicians’ willingness to invest in respectful, patient, and norm-consistent interaction with colleagues, thereby increasing the likelihood of enacted discourtesy toward members of another professional group (Lewis, 2023; Shantz et al., 2014). The finding of partial mediation would be especially meaningful because it would suggest that the relational risks of Gen-AI-assisted collaboration in hospitals are not reducible to a single pathway. Rather, the effect appears to unfold through both immediate interactional tension and a more internalized process of work alienation. Therefore:

*H4*: Work alienation mediates the relationship between Gen-AI-assisted clinical work collaboration and instigated interprofessional incivility.

### 2.4 The moderating role of trait emotional intelligence

Yet not all clinicians respond to alienating work conditions in the same way. Trait emotional intelligence (trait EI) offers one potential explanation for this variation (Ma and Liu, 2019). Trait EI refers to a constellation of emotion-related self-perceptions concerning how effectively individuals believe they can understand, regulate, express, and utilize emotions in everyday life (Petrides and Furnham, 2003; Petrides et al., 2007; Petrides et al., 2016). Trait EI was selected as the focal moderator not because it is easily modified in the short term, but because it provides a theoretically meaningful and relatively stable individual-difference lens for explaining why clinicians may respond differently to the same alienating work condition (Zadorozhny et al., 2024). As a personality trait located at the lower levels of personality hierarchies, trait EI exhibits temporal stability with test–retest correlations ranging from 0.67 to 0.80 over intervals spanning 1 month to 4 years, making it particularly suitable for identifying boundary conditions of strain processes in observational research designs (Petrides and Furnham, 2003; Zadorozhny et al., 2024).

In the present study, trait EI is operationalized through the Wong and Law Emotional Intelligence Scale (WLEIS), which conceptualizes emotional intelligence through four related but distinct dimensions: self-emotion appraisal, others’ emotion appraisal, use of emotion, and regulation of emotion (Wong and Law, 2002). This distinction is important because the focal moderation occurs at the point where a negative internal work state—namely, work alienation—is translated into outward interprofessional behavior. In other words, the key issue is not simply whether clinicians feel alienated, but whether they can accurately recognize, interpret, manage, and redirect the emotional consequences of alienation before these consequences spill over into discourteous conduct toward colleagues (Ma and Liu, 2019).

This moderating logic is also consistent with JD-R theory. Within the JD-R framework, personal resources help individuals cope with job-related strain and reduce the likelihood that adverse work conditions culminate in maladaptive outcomes (Bakker and Demerouti, 2017; Bakker et al., 2023). Trait EI can be understood as such a personal resource because it facilitates emotional awareness, adaptive self-regulation, and interpersonal sensitivity under demanding work conditions (Powell et al., 2024). Emerging evidence further indicates that trait EI functions as a protective factor against burnout and turnover intention among nurses, suggesting that it helps clinicians manage the emotional demands of healthcare work without disengaging from their professional responsibilities (Galanis et al., 2024). From this perspective, trait EI should weaken the positive relationship between work alienation and instigated interprofessional incivility by reducing the likelihood that alienation-driven frustration, detachment, or resentment will be externalized in cross-professional interaction (Petrides et al., 2016; Powell et al., 2024). Recent evidence indicates that EI mediates the relationship between technostress and burnout among critical care nurses, suggesting that the emotional demands of technology-intensive healthcare work may operate through both resource-depletion and resource-preservation pathways (Shaban et al., 2025). More specifically, self-emotion appraisal should help clinicians recognize frustration and detachment before these feelings are displaced onto colleagues. Others’ emotion appraisal should increase sensitivity to emotional cues in interprofessional exchanges. Use of emotion should facilitate constructive coping, whereas regulation of emotion should inhibit discourteous expression when strain is high. The Wong and Law Emotional Intelligence Scale has also demonstrated validity and reliability in nursing and healthcare-related samples (Park and Yu, 2021; Shah, 2022). Accordingly, the present study proposes the following hypotheses:

*H5a*: Self-emotion appraisal moderates the relationship between work alienation and instigated interprofessional incivility, such that the relationship is weaker when self-emotion appraisal is higher.

*H5b*: Others’ emotion appraisal moderates the relationship between work alienation and instigated interprofessional incivility, such that the relationship is weaker when others’ emotion appraisal is higher.

*H5c*: Use of emotion moderates the relationship between work alienation and instigated interprofessional incivility, such that the relationship is weaker when use of emotion is higher.

*H5d*: Regulation of emotion moderates the relationship between work alienation and instigated interprofessional incivility, such that the relationship is weaker when regulation of emotion is higher.

*H6*: Trait EI moderates the relationship between work alienation and instigated interprofessional incivility, such that the relationship is weaker when trait EI is higher.

**Figure 1:**
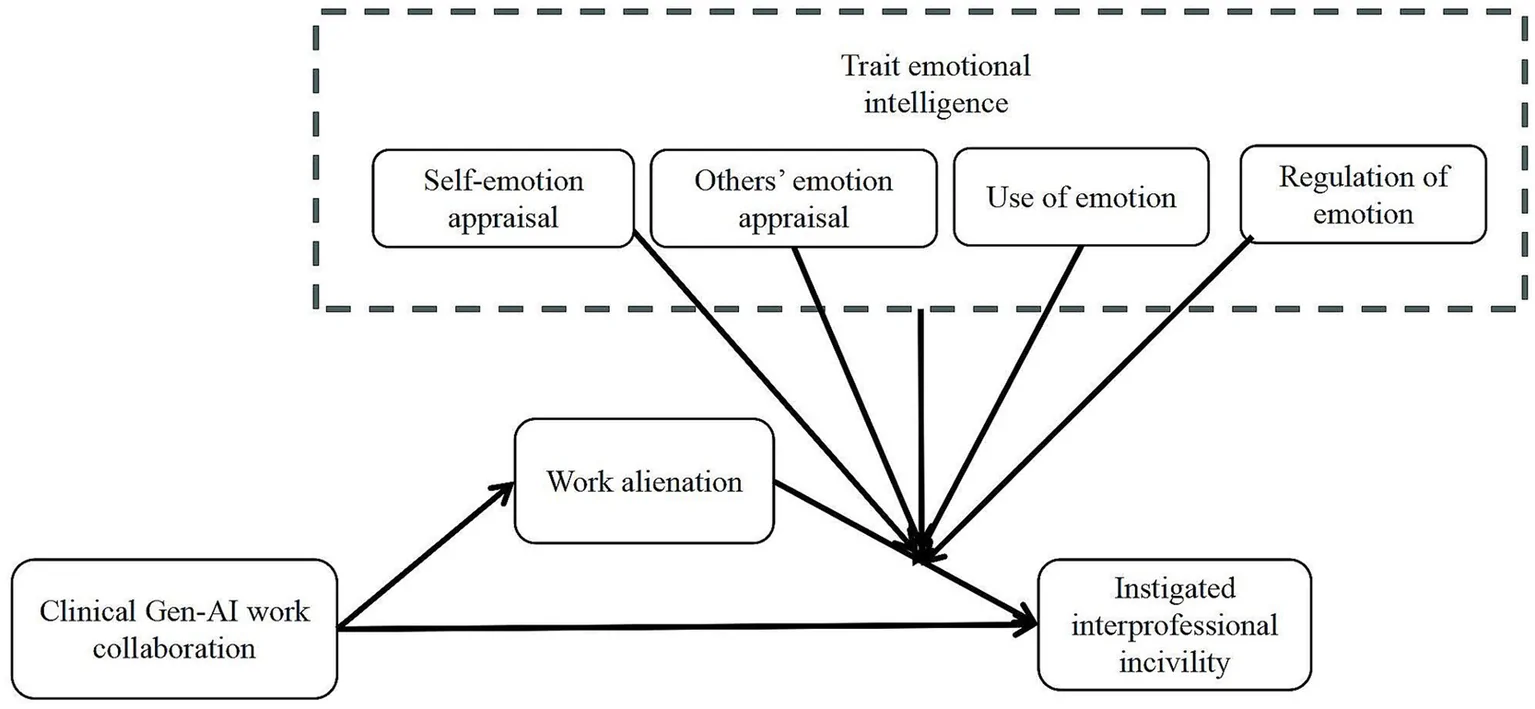
Conceptual framework.

Finally, a unified conceptual framework is constructed, as in Figure 1, illustrating the proposed model: Gen-AI-assisted clinical work collaboration is positively associated with instigated interprofessional incivility both directly (H1) and indirectly through work alienation (H2–H4), while trait EI and its four dimensions condition the alienation–incivility relationship (H5a–H6). For additional clarity, Supplementary Table S1 summarizes the key supporting literature, hypotheses, and corresponding empirical tests for H1–H6, while Figure 1 presents the integrated conceptual model.

## 3 Materials and methods

### 3.1 Data collection and sampling

To test the proposed model, this study collected data from physicians and nurses at Xiamen University Affiliated Zhongshan Hospital in China using a three-wave time-lagged survey design. This design was adopted because the focal relationships in the study are temporally ordered: Gen-AI-assisted clinical work collaboration is theorized to influence a subsequent psychological state, namely work alienation, which in turn is associated with later enacted interprofessional behavior. In addition, temporal separation is widely recommended as a procedural remedy for reducing common method variance (CMV) in self-report research because it weakens respondents’ tendency to rely on prior answers when completing later measures (Yao and Xu, 2024). Compared with a single-wave survey, a three-wave time-lagged design is better suited to capturing the sequential nature of the proposed mechanism while remaining feasible in a hospital setting (Podsakoff et al., 2003; Podsakoff et al., 2012).

Xiamen University Affiliated Zhongshan Hospital provides an appropriate context for examining the present model because physicians and nurses in tertiary hospitals work under sustained time pressure, emotional labor, and interprofessional coordination demands, while increasingly encountering Gen-AI-assisted tools in clinical documentation, information synthesis, communication drafting, and decision preparation. To improve access to eligible respondents, the questionnaires were distributed online through Wenjuanxing (WJX) and data collection was facilitated by the co-author who is a staff member at Xiamen University Affiliated Zhongshan Hospital, which enabled direct access to eligible clinical departments, professional WeChat and QQ groups for physicians and nurses, and internal hospital communication channels that would not otherwise be accessible to external researchers. This internal connection was instrumental in achieving a satisfactory response rate and ensuring that the sample was drawn from the target population of actively practicing clinicians.

The questionnaire included screening items regarding respondents’ hospital affiliation, occupational identity, demographic characteristics, and current AI use status. Specific screening questions verified whether the respondent was currently working as a physician or nurse at Xiamen University Affiliated Zhongshan Hospital (“Are you currently working as a physician or nurse at Xiamen University Affiliated Zhongshan Hospital?”), whether they were actively engaged in clinical or nursing work involving direct patient contact (“Are you currently engaged in clinical or nursing work involving direct patient contact?”), and whether they had used AI-assisted tools in their clinical or nursing work within the past month (“Have you used AI-assisted tools in your clinical or nursing work within the past month?”). Responses failing any screening item were excluded from analysis. Participation was entirely voluntary.

Before formal data collection, a pilot study was conducted in January 2026 with 95 respondents using QR code linking to the online survey (hosted on WJX). The pilot study was used to assess the clarity, contextual appropriateness, and preliminary reliability of the questionnaire. The pilot data were analyzed using SPSS 27.0 and AMOS 24.0. Exploratory factor analysis (EFA) was performed, and the results showed that the overall Cronbach’s alpha of the questionnaire was 0.914, indicating high internal consistency. The Kaiser–Meyer–Olkin (KMO) value was 0.810, which exceeded the recommended threshold of 0.70 and suggested that the data were appropriate for factor analysis. The pilot results further indicated that the 28 focal items showed acceptable reliability. Based on the pilot feedback and statistical results, minor wording revisions were made to improve readability and contextual fit for Chinese healthcare professionals while preserving the original meaning of the source scales. In addition, the translated questionnaire was reviewed by a panel of five experts (two human resource management scholars, two hospital practice specialists, and one artificial intelligence researcher). Discrepancies between translations were resolved through consensus discussion.

The formal data collection was conducted between February and March 2026. At Time 1, participants completed the measures of Gen-AI-assisted clinical work collaboration, trait EI, and demographic variables. Two weeks later, at Time 2, the same participants completed the measure of work alienation. After another two-week interval, at Time 3, participants completed the measure of instigated interprofessional incivility. The two-week interval between adjacent waves was adopted to preserve temporal separation among the focal constructs while maintaining respondent participation across the three waves; similar spacing has also been used in recent time-lagged healthcare research (Luo et al., 2025). To match responses across the three waves while preserving anonymity, participants were instructed to generate a personal identification code at each time point using the following formula: the first letter of their surname combined with their birth month and the last digit of their employee ID number. This multi-factor combination produced a distinctive code for matching purposes while containing no personally identifying information. Participants recorded this code privately and entered it at the beginning of each wave’s questionnaire. This procedure allowed the research team to link responses across waves without collecting personally identifying information.

At Time 1, 535 submissions were received, of which 514 were retained as valid responses after screening. At Time 2, 542 submissions were received, of which 497 could be successfully matched to the valid Time 1 cases. At Time 3, 533 submissions were received, of which 468 could be successfully matched across all three waves. After excluding incomplete and unmatched responses, the final sample consisted of 468 valid participants, and these matched cases were retained for the subsequent analyses. These matching and exclusion procedures ensured that only complete and cross-wave matched responses from eligible target respondents were retained for the final analysis. Across the three waves, 46 participants (9.0% of the Time 1 valid sample) did not complete all waves. Attrition was primarily attributable to work scheduling conflicts, leave absences, and voluntary withdrawal.

Before participating, all respondents were informed of the purpose of the study and were assured that their responses would be used only for academic research. They were also informed that participation was voluntary and that they could withdraw at any stage without penalty. Confidentiality and anonymity were emphasized throughout the data collection process to encourage honest responses, particularly because some items concerned emotionally sensitive work experiences and enacted interpersonal behavior.

### 3.2 Measures

All constructs were measured using established scales and adapted to the Chinese healthcare context and the present three-wave time-lagged design. The original English scales were translated and backtranslated, reviewed by experts in human resource management, hospital practice, and artificial intelligence, and slightly revised based on pilot feedback while preserving their original meaning. Unless otherwise noted, all focal constructs were assessed on five-point Likert-type scales ranging from 1 = strongly disagree to 5 = strongly agree.

Gen-AI-assisted clinical work collaboration (CGW) was measured at Time 1 with five items adapted from Hai et al. (2025) and Kong et al. (2023). Following Anthony et al.’s (2023) conceptualization of human–AI collaboration as a system-level counterpart relationship, this variable was operationalized as the degree to which clinicians experience Gen-AI tools as active participants in their clinical work processes. The scale captures AI involvement across multiple work facets, including problem-solving, information processing, and task execution. A sample item is: “In my work, AI-assisted tools are involved in my clinical/nursing problem-solving.” Work alienation was measured at Time 2 with three items adapted from Hai et al. (2025) and Shantz et al. (2014). A sample item is: “I feel emotionally detached from my work.” Instigated interprofessional incivility was measured at Time 3 using the instigated incivility subscale of the Straightforward Incivility Scale (Leiter and Day, 2013), with wording adapted to fit interprofessional hospital interactions. A sample item is: “I respond impatiently to members of the other professional group.” Trait EI was measured at Time 1 using the 16-item WLEIS, which comprises self-emotion appraisal, others’ emotion appraisal, use of emotion, and regulation of emotion (Wong and Law, 2002). A sample item is: “I have a clear understanding of my own emotions.” The four WLEIS dimensions were used in the primary moderation analyses, while an overall trait EI index was additionally computed for supplementary analysis. The questionnaire contained 28 focal items and 11 non-focal items, yielding 39 items in total. The non-focal items included screening items, matching-code items, and demographic questions.

### 3.3 Analytical strategy

The data were analyzed using SPSS 27.0, AMOS 24.0, and PROCESS 4.0 for SPSS. First, SPSS 27.0 was used for preliminary analyses, including descriptive statistics and Harman’s single-factor test for CMV. Second, AMOS 24.0 was used to assess the measurement model and test the structural relationships among the focal constructs. Reliability and convergent validity were evaluated using standardized factor loadings, composite reliability (CR), and average variance extracted (AVE), whereas discriminant validity was assessed by comparing the square roots of the AVE values with the inter-construct correlations. Confirmatory factor analysis (CFA) was then conducted to evaluate model fit, followed by structural path analysis to test H1, H2, and H3. Third, H4 was tested using bootstrap mediation analysis with 5,000 resamples and 95% confidence intervals (Hayes, 2017). The mediating effect was considered significant when the confidence interval of the indirect effect did not include zero. Finally, H5a–H5d and H6 were tested using PROCESS 4.0 with 95% confidence intervals. The four dimensions of trait EI were first examined as separate moderators of the relationship between work alienation and instigated interprofessional incivility, followed by a supplementary analysis using the overall trait EI index.

## 4 Results

### 4.1 Sample characteristics

The final sample consisted of 468 valid respondents. In terms of gender, 48.1% of the respondents were male and 51.9% were female. Regarding age, the largest group was 36–40 years old, accounting for 30.1% of the sample. In terms of educational attainment, respondents with a master’s degree accounted for the largest proportion (36.1%), followed by those with a bachelor’s degree (31.0%). With respect to years of experience in the healthcare profession, the largest group had worked for 3–5 years (27.3%), followed by those with 1–3 years of experience (21.8%). In terms of occupation, 54.7% of the respondents were nurses and 45.3% were physicians.

### 4.2 Common method variance, reliability, validity, and measurement model fit

Common Method Variance was assessed using Harman’s single-factor test, following Podsakoff et al. (2003). Seven factors had eigenvalues greater than 1, and the first unrotated factor accounted for 33.381% of the total variance, below the 40% threshold, suggesting that CMV is unlikely to be a serious concern.

The reliability, validity, and overall fit of the measurement model were then assessed using AMOS 24.0. The KMO value was 0.917, indicating that the data were appropriate for factor analysis. As shown in Table 1, all standardized factor loadings were significant at *p* < 0.001 and ranged from 0.778 to 0.896, indicating satisfactory item reliability. In addition, the CR values for all constructs ranged from 0.883 to 0.921, exceeding the recommended threshold of 0.70, and the AVE values ranged from 0.657 to 0.717, exceeding the recommended threshold of 0.50. These results indicate satisfactory internal consistency and convergent validity.

**Table 1:** Reliability and convergent validity.

Construct	Item	Unstd.	S.E.	*p*	Std.	CR	AVE
Clinical Gen-AI work collaboration (CGW)	CGW1	1	—	—	0.837	0.921	0.700
	CGW2	1.024	0.048	***	0.822		
	CGW3	1.057	0.049	***	0.822		
	CGW4	1.073	0.048	***	0.844		
	CGW5	1.079	0.047	***	0.859		
Work alienation (DWA)	DWA1	1	—	—	0.879	0.883	0.717
	DWA2	0.958	0.042	***	0.866		
	DWA3	0.919	0.045	***	0.792		
Self-emotion appraisal (SEA)	SEA1	1	—	—	0.894	0.901	0.696
	SEA2	0.879	0.041	***	0.794		
	SEA3	0.925	0.038	***	0.861		
	SEA4	0.875	0.042	***	0.783		
Others’ emotion appraisal (OEA)	OEA1	1	—	—	0.848	0.891	0.671
	OEA2	1.018	0.049	***	0.831		
	OEA3	0.895	0.047	***	0.780		
	OEA4	0.946	0.046	***	0.817		
Use of emotion (UOE)	UOE1	1	—	—	0.798	0.884	0.657
	UOE2	1.037	0.058	***	0.776		
	UOE3	1.059	0.055	***	0.826		
	UOE4	1.078	0.055	***	0.841		
Regulation of emotion (ROE)	ROE1	1	—	—	0.839	0.894	0.679
	ROE2	0.958	0.049	***	0.788		
	ROE3	0.995	0.048	***	0.832		
	ROE4	0.998	0.047	***	0.836		
Instigated interprofessional incivility (III)	III1	1	—	—	0.828	0.887	0.664
	III2	0.984	0.052	***	0.777		
	III3	1.032	0.051	***	0.818		
	III4	1.068	0.051	***	0.836		

Unstd., unstandardized factor loading; S.E., standard error; Std., standardized factor loading; CR, composite reliability; AVE, average variance extracted. ***p < 0.001.

Discriminant validity was also supported. As shown in Table 2, the square roots of the AVE values for each construct exceeded the corresponding inter-construct correlations, indicating that the focal constructs were empirically distinct from one another.

**Table 2:** Discriminant validity.

Construct	ROE	DWA	UOE	III	OEA	SEA	CGW
ROE	**0.819**						
DWA	−0.340	**0.847**					
UOE	0.429	−0.282	**0.811**				
III	−0.506	0.572	−0.497	**0.815**			
OEA	0.214	−0.194	0.281	−0.387	**0.819**		
SEA	0.251	−0.171	0.324	−0.394	0.335	**0.834**	
CGW	−0.349	0.541	−0.337	0.707	−0.287	−0.260	**0.837**

Bold diagonal values are the square roots of AVE (Fornell and Larcker, 1981). Off-diagonal values are inter-construct correlations.

The overall fit of the measurement model was satisfactory. The model fit the data well: *χ*^2^ = 359.019, *df* = 329, *χ*^2^/*df* = 1.091, GFI = 0.949, AGFI = 0.937, CFI = 0.990, TLI = 0.996, and RMSEA = 0.014. All indices met or exceeded the commonly recommended thresholds, indicating that the measurement model demonstrated a good fit to the data.

### 4.3 Structural model results

**Figure 2:**
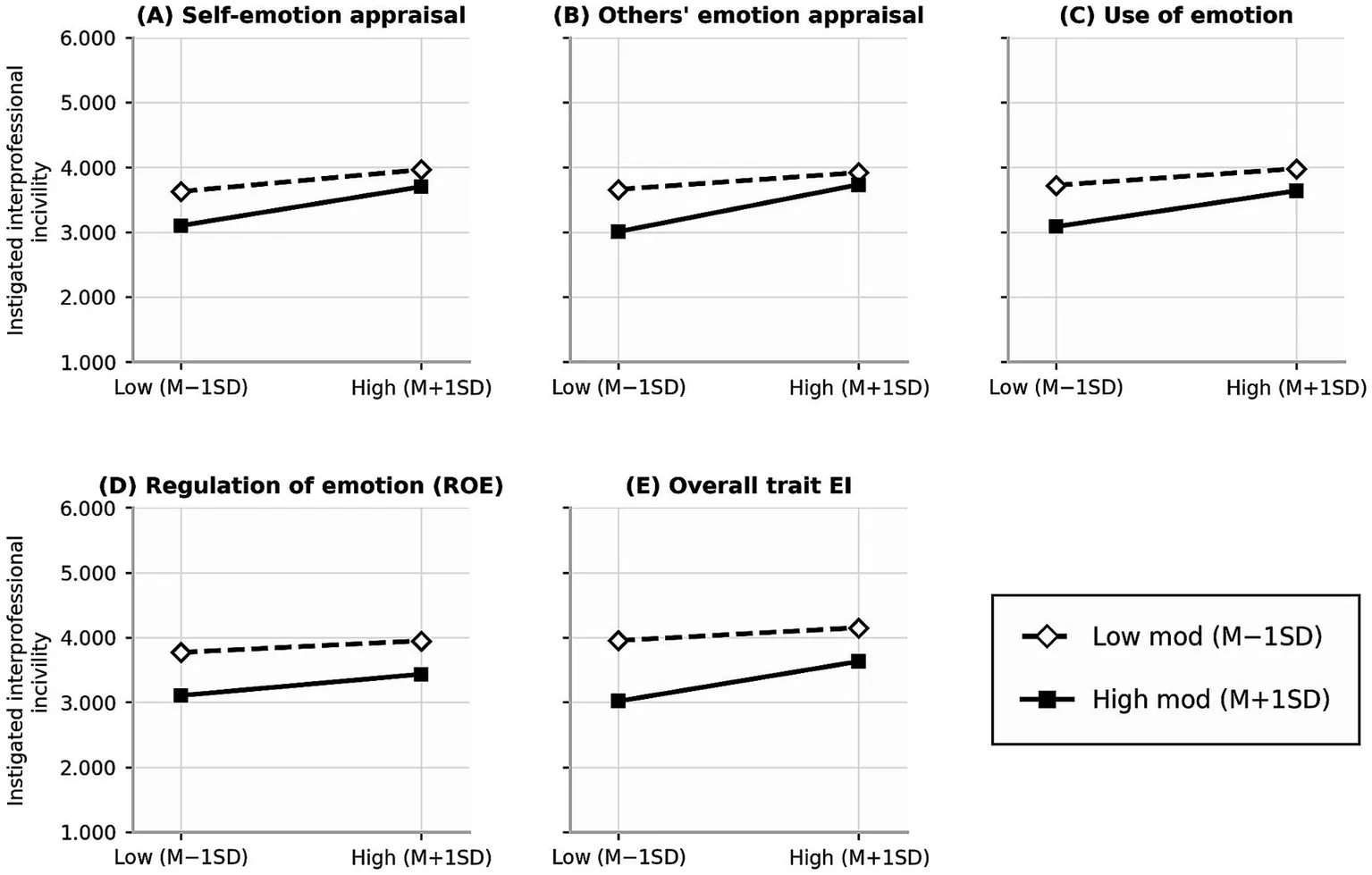
Figure of the moderating effects. **(A)** Self-emotion appraisal (SEA), **(B)** Others’ emotion appraisal (OEA), **(C)** Use of emotion (UOE), **(D)** Regulation of emotion (ROE), **(E)** Overall trait emotional intelligence (TEI). DWA = work alienation. Dashed line = low moderator (1 SD below the mean); solid line = high moderator (1 SD above the mean).

For ease of interpretation, the empirical tests are organized to correspond directly to the conceptual model and hypotheses. Table 3 reports the direct-effect tests for H1–H3, Table 4 reports the mediation test for H4, and Table 5, together with Figure 2, reports the moderation tests for H5a–H6.

**Table 3:** Direct effects and hypothesis testing.

Relationship	Hypothesis	Unstd.	S.E.	*Z* value	*p*	Std. (*β*)	Result
CGW → III	H1	0.479	0.044	10.917	***	0.562	Supported
CGW → DWA	H2	0.602	0.054	11.171	***	0.541	Supported
DWA → III	H3	0.205	0.037	5.542	***	0.268	Supported

***p < 0.001.

**Table 4:** Bootstrap mediation results.

Parameter	Effect value	SE	95% confidence interval Boot LLCI	95% confidence interval Boot ULCI
Direct effect (CGW → III)	0.562	0.043	0.479	0.645
Indirect effect (CGW → DWA → III)	0.145	0.029	0.088	0.203
Total effect	0.707	0.028	0.650	0.757

Bootstrap resamples = 5,000. LLCI, lower-level confidence interval; ULCI, upper-level confidence interval.

**Table 5:** Moderation results.

Variable	Coeff.	SE	*t*	*p*	LLCI	ULCI
Constant	2.155	0.124	17.406	***	1.911	2.398
CGW	0.420	0.035	12.022	***	0.351	0.488
DWA	0.208	0.030	6.835	***	0.148	0.268
SEA	−0.187	0.029	−6.384	***	−0.245	−0.130
DWA × SEA	0.056	0.024	2.297	0.022	0.008	0.104
*R* ^2^	0.505					
Constant	2.138	0.125	17.149	***	1.893	2.383
CGW	0.426	0.035	12.125	***	0.357	0.495
DWA	0.197	0.031	6.412	***	0.137	0.258
OEA	−0.182	0.032	−5.752	***	−0.244	−0.120
DWA × OEA	0.080	0.028	2.886	0.004	0.026	0.135
*R* ^2^	0.498					
Constant	2.215	0.123	18.054	***	1.974	2.456
CGW	0.405	0.034	11.745	***	0.337	0.472
DWA	0.180	0.030	5.979	***	0.121	0.240
UOE	−0.246	0.032	−7.766	***	−0.308	−0.183
DWA × UOE	0.067	0.028	2.435	0.015	0.013	0.121
*R* ^2^	0.522					
Constant	2.208	0.124	17.833	***	1.965	2.451
CGW	0.409	0.035	11.818	***	0.341	0.477
DWA	0.158	0.031	5.069	***	0.097	0.219
ROE	−0.240	0.032	−7.404	***	−0.303	−0.176
DWA × ROE	0.076	0.030	2.542	0.011	0.017	0.135
*R* ^2^	0.517					
Constant	2.406	0.119	20.260	***	2.173	2.639
CGW	0.351	0.033	10.511	***	0.285	0.417
DWA	0.159	0.029	5.546	***	0.103	0.215
TEI	−0.490	0.045	−10.983	***	−0.578	−0.402
DWA × TEI	0.101	0.036	2.836	0.005	0.031	0.171
*R* ^2^	0.572					

***p < 0.001.

#### 4.3.1 Direct effects and hypothesis testing

As shown in Table 3, the structural model results indicated that CGW had a significant positive direct effect on III (*β* = 0.562, *p* < 0.001), thereby supporting H1. CGW also showed a significant positive effect on DWA (*β* = 0.541, *p* < 0.001), thereby supporting H2. In addition, DWA had a significant positive effect on III (*β* = 0.268, *p* < 0.001), thereby supporting H3.

#### 4.3.2 Mediation effect of work alienation

As shown in Table 4, the indirect effect of CGW on III through DWA was 0.145, with a 95% bootstrap CI ranging from 0.088 to 0.203. Because the CI did not include zero, the mediating effect of DWA was supported. At the same time, the direct effect of CGW on III remained significant [effect = 0.562, 95% CI (0.479, 0.645)], indicating that the relationship between CGW and III was not fully explained by DWA alone. The total effect of CGW on III was 0.707, with a 95% CI of [0.650, 0.757], further confirming that Gen-AI-assisted clinical work collaboration exerted a substantial overall influence on instigated interprofessional incivility. These results support H4 and indicate that work alienation functions as a significant but partial mediating mechanism linking Gen-AI-assisted clinical work collaboration to instigated interprofessional incivility. This pattern is theoretically important because it indicates that the relational risks of Gen-AI-assisted clinical work collaboration are not reducible to a single mechanism. Instead, the findings suggest both a direct relational-disruption pathway and an indirect alienation-based pathway through which technologically mediated work conditions may foster discourteous interprofessional conduct in hospital settings.

#### 4.3.3 Moderating effects

The moderation results are presented in Table 5, and the interaction patterns are illustrated in Figure 2. Across all models, CGW remained a significant positive predictor of III, suggesting that the direct effect identified in Section 3.3.1 remained robust even after the inclusion of the interaction terms. At the same time, the focal moderators—SEA, OEA, UOE, ROE, and overall TEI—each showed significant main effects on III, indicating that higher emotional intelligence was generally associated with lower levels of instigated interprofessional incivility.

More importantly, the interaction terms between DWA and each moderator were all statistically significant. Specifically, the interaction effects were significant for SEA (*β* = 0.056, *p* = 0.022), OEA (*β* = 0.080, *p* = 0.004), UOE (*β* = 0.067, *p* = 0.015), ROE (*β* = 0.076, *p* = 0.011), and overall TEI (*β* = 0.101, *p* = 0.005). These findings indicate that the relationship between work alienation and instigated interprofessional incivility varied across levels of emotional intelligence. However, as shown by the positive signs of the interaction terms and the plotted interaction patterns in Figure 2, the direction of these moderating effects was opposite to that hypothesized. Rather than weakening the positive relationship between DWA and III, higher levels of SEA, OEA, UOE, ROE, and overall TEI appeared to strengthen the extent to which work alienation was associated with instigated interprofessional incivility. Therefore, although significant moderation effects were observed, H5a, H5b, H5c, H5d, and H6 were not supported in the hypothesized direction.

## 5 Discussion

### 5.1 Main findings

This study examined how Gen-AI-assisted clinical work collaboration shapes instigated interprofessional incivility among physicians and nurses in the hospital context. Drawing on human–AI collaboration research and the strain process within JD-R theory, the study tested a model in which Gen-AI-assisted clinical work collaboration influences instigated interprofessional incivility both directly and indirectly through work alienation, while trait EI and its four dimensions condition the alienation–incivility relationship (Anthony et al., 2023; Raisch and Krakowski, 2021). The findings support the central claim that Gen-AI-assisted clinical work collaboration is not merely an efficiency-enhancing arrangement in healthcare work but may also generate relational and psychological costs that affect clinicians’ interprofessional conduct (Hai et al., 2025; Lewis, 2023).

More specifically, the results showed that Gen-AI-assisted clinical work collaboration was positively associated with both instigated interprofessional incivility and work alienation, and that work alienation was positively associated with instigated interprofessional incivility. The significant direct effect of CGW on III is consistent with the direct relational disruption logic developed in Section 1, which argued that Gen-AI-assisted clinical work collaboration may affect interaction patterns in addition to altering employees’ relationship with their work. More specifically, when Gen-AI becomes involved in documentation, information synthesis, and decision preparation, communication rhythm, responsibility boundaries, and coordination processes may become more strained, thereby increasing the likelihood of low-intensity discourteous conduct toward interprofessional colleagues (Anthony et al., 2023; Bakker and Demerouti, 2017). In this sense, the present finding extends human–AI collaboration research by showing that Gen-AI-assisted collaboration may carry not only task-related implications but also immediate relational consequences in high-pressure hospital settings.

The significant positive relationship between CGW and DWA is in line with the argument developed in Section 1, which drew on human–AI collaboration research and JD-R theory to suggest that Gen-AI-assisted collaboration may reduce employees’ sense of control, authorship, and meaningful engagement while simultaneously introducing additional monitoring, verification, and accountability demands (Hai et al., 2025; Raisch and Krakowski, 2021). Thus, the present result supports the view that Gen-AI-assisted clinical work collaboration is not experienced merely as an efficiency-enhancing arrangement but may also become a psychologically distancing work condition that heightens alienation. In addition, the positive effect of DWA on III is consistent with the argument that work alienation is not merely an attitudinal response to work but a psychologically distancing condition that weakens employees’ willingness to invest in respectful, patient, and norm-consistent interaction with colleagues (Chiaburu et al., 2014; Shantz et al., 2014). It also accords with the incivility literature, which indicates that low-intensity disrespect is more likely to emerge under conditions of strain, weakened relational investment, and diminished interpersonal restraint (Blau and Andersson, 2005; Lewis, 2023; Park and Martinez, 2022). In the hospital context, where interprofessional collaboration depends heavily on respectful and coordinated communication, such alienation-driven deterioration in relational investment appears especially consequential.

The mediation results further reinforce the central argument of the present study. Work alienation functioned as a significant but partial mediating mechanism linking Gen-AI-assisted clinical work collaboration to instigated interprofessional incivility. Consistent with human–AI collaboration research and JD-R theory, Gen-AI-assisted clinical work collaboration may weaken clinicians’ sense of control, authorship, and meaningful engagement while simultaneously increasing monitoring, verification, and accountability demands, thereby heightening work alienation (Raisch and Krakowski, 2021). In turn, work alienation may erode clinicians’ willingness to invest in respectful, patient, and norm-consistent interaction with colleagues, thereby increasing the likelihood of enacted discourtesy toward members of another professional group (Lewis, 2023; Shantz et al., 2014). The finding of partial mediation is especially meaningful because it suggests that the relational risks of Gen-AI-assisted collaboration in hospitals are not reducible to a single pathway. Rather, as proposed in Section 1, the effect appears to unfold through both immediate interactional tension and a more internalized process of work alienation, indicating that Gen-AI-assisted clinical work collaboration may influence instigated interprofessional incivility both directly and indirectly through work alienation (Anthony et al., 2023; Bakker et al., 2023; Hai et al., 2025). In substantive terms, this dual-pathway pattern offers a more nuanced understanding of how Gen-AI-assisted clinical work collaboration may contribute to interprofessional misconduct in healthcare settings, where respectful, coordinated communication remains central to effective teamwork and service quality (Lewis, 2023). In other words, the partial mediation finding suggests that work alienation explains an important part—but not the entirety—of how Gen-AI-assisted clinical work collaboration translates into interprofessional misconduct, thereby reinforcing the view that both relational disruption and psychological distancing are integral to the dark side of technologically mediated hospital work.

The moderation results suggest that the role of trait EI in technologically mediated healthcare work is more complex than originally hypothesized. Although trait EI and its four dimensions showed significant main effects, their interaction effects with work alienation on instigated interprofessional incivility were significant in the opposite direction from that predicted. Rather than buffering the interpersonal consequences of work alienation, higher levels of trait EI appeared to strengthen the alienation–incivility relationship under the present conditions. This pattern is theoretically noteworthy because it departs from the buffering logic proposed in Section 1. In the literature review, trait EI was conceptualized as a personal resource within the JD-R framework, one that should help clinicians recognize, manage, and redirect alienation-related affect before it spills over into discourteous conduct (Bakker and Demerouti, 2017; Bakker et al., 2023; Petrides et al., 2016; Wong and Law, 2002).

More specifically, the present study proposed that SEA, OEA, UOE, ROE, and overall TEI should reduce the likelihood that work alienation would be translated into enacted disrespect by improving emotional recognition, interpersonal sensitivity, adaptive utilization of affect, and emotional regulation (Ma and Liu, 2019; Petrides et al., 2016; Wong and Law, 2002). The present findings suggest a more complex pattern in the hospital context. Rather than uniformly absorbing the negative interpersonal implications of alienation, emotional intelligence may under some conditions heighten clinicians’ awareness of relational tension, role strain, and emotionally charged interprofessional dynamics. In such a context, individuals higher in emotional perception and emotional utilization may become more behaviorally responsive to alienation-related strain instead of simply disengaging from it. Thus, trait EI did not operate in the hypothesized buffering direction, suggesting that its role in technologically mediated healthcare work may be more context-dependent and ambivalent than originally expected (Lewis, 2023; Reeves et al., 2017).

Taken together, these findings support a more integrated understanding of the proposed model. Gen-AI-assisted clinical work collaboration appears to shape instigated interprofessional incivility through both direct relational effects and indirect alienation-based effects, while emotional resources operate as context-dependent rather than uniformly protective boundary conditions. This integrated pattern helps reduce remaining interpretive gaps in the model by showing that the darker side of technologically mediated hospital work is best understood as the combined result of interactional disruption, psychological distancing, and contingent emotional dynamics.

### 5.2 Theoretical implications

Theoretically, this study advances understanding of Gen-AI-assisted work in healthcare by integrating human–AI collaboration research with the strain process within JD-R theory to explain the darker side of Gen-AI-assisted clinical work collaboration. Whereas prior research has mainly emphasized the productivity, decision-support, and efficiency benefits of human–AI collaboration, the present findings show that Gen-AI-assisted clinical work collaboration may also generate meaningful psychological and interpersonal costs in hospital work. More specifically, the results suggest that Gen-AI-assisted clinical work collaboration is positively associated with instigated interprofessional incivility both directly and indirectly through work alienation. In this sense, the study extends human–AI collaboration research from a primarily task-oriented perspective to one that also captures relational and behavioral outcomes in high-stakes professional settings (Anthony et al., 2023; Kong et al., 2023). The study also contributes to JD-R theory by showing that Gen-AI-assisted clinical work collaboration may operate as a technologically mediated work condition that introduces new cognitive, emotional, and role-related demands, which in turn foster work alienation and downstream interpersonal consequences (Bakker and Demerouti, 2017; Bakker et al., 2023). More specifically, the present study contributes by isolating the demand-inducing side of Gen-AI-assisted collaboration and showing how this underexamined strain pathway may foster work alienation and downstream interpersonal consequences in hospital work.

In addition, by identifying work alienation as a significant but partial mediating mechanism, the study contributes to the work alienation and incivility literatures and offers a more nuanced explanation of how technologically mediated work conditions may translate into discourteous interprofessional conduct (Andersson and Pearson, 1999; Blau and Andersson, 2005; Chiaburu et al., 2014). Finally, the unexpected moderation findings refine current understanding of trait EI by suggesting that, in emotionally charged and highly interdependent clinical environments, emotional resources may not always function as uniformly protective factors but may instead operate in a more context-dependent and ambivalent manner (Petrides et al., 2016; Wong and Law, 2002).

Moreover, the inclusion of trait EI contributes theoretically by identifying a stable boundary condition that helps explain heterogeneity in clinicians’ behavioral responses to work alienation under technologically mediated work conditions. The unexpected direction of the moderating effect—whereby higher trait EI strengthened rather than weakened the alienation–incivility relationship—challenges the default assumption that personal resources uniformly buffer the negative consequences of work strain (Szczygieł and Mikołajczak, 2018). This pattern supports a more context-dependent view of the JD-R model’s moderating mechanism, suggesting that the protective function of emotional resources may be contingent on situational factors such as professional norms, relational interdependence, and face concerns in hospital environments. In this sense, the moderation findings do not simply contradict the proposed model; rather, they refine it by showing that the behavioral consequences of work alienation in hospital settings may depend not only on the presence of emotional resources, but also on how those resources operate within emotionally charged and highly interdependent professional interactions.

### 5.3 Practical implications

This study offers several practical implications for hospital management, particularly for Xiamen University Affiliated Zhongshan Hospital and other Chinese hospitals undergoing rapid Gen-AI integration. First, the findings suggest that Gen-AI-assisted tools should not be evaluated solely in terms of efficiency, speed, or decision-support convenience. Although Gen-AI-assisted clinical work collaboration may improve workflow performance, it may also generate unintended psychological and interpersonal costs. Hospital leaders should therefore treat Gen-AI adoption not only as a technical innovation issue, but also as a work design and interprofessional coordination issue.

Second, the finding that work alienation partially mediated the relationship between Gen-AI-assisted clinical work collaboration and instigated interprofessional incivility indicates that the relational risks of AI-supported work arise not only from immediate communication strain, but also from clinicians’ psychological distancing from their work. Hospitals should therefore monitor whether AI-supported workflows weaken clinicians’ sense of authorship, control, and meaningful engagement, and should introduce AI in ways that preserve professional judgment and final interpretive authority (Raisch and Krakowski, 2021). Third, hospitals should establish clearer responsibility boundaries and communication protocols in AI-assisted clinical work. Because Gen-AI-assisted collaboration may introduce additional monitoring, verification, and accountability demands, ambiguity regarding who is responsible for reviewing outputs and making final decisions may intensify interprofessional tension. Embedding AI governance into routine workflow protocols may therefore help reduce discourteous conduct across professional groups.

Finally, the moderation results carry practical implications for how hospitals should approach the relational risks of AI-assisted work. The practical value of trait EI in the present study lies less in the assumption that this stable characteristic can be easily changed in the short term, and more in its usefulness for identifying differential vulnerability to alienation-related interpersonal strain. Hospitals may use this insight to improve risk awareness, team support, communication protocols, and managerial attention to departments where technologically mediated strain is likely to spill over into interprofessional misconduct. In this sense, the findings do not suggest that trait EI itself should be treated as a simple intervention target; rather, they indicate that emotional differences among clinicians should be considered when designing workflow support, conflict-prevention strategies, and communication-focused interventions. More effective approaches are likely to require a combination of workflow redesign, accountability clarification, communication support, and managerial attention to department-level strain. More broadly, the study suggests that successful Gen-AI integration in healthcare depends not only on whether the technology improves efficiency, but also on whether it preserves meaningful work engagement, professional responsibility, and respectful interprofessional collaboration (Lewis, 2023; Reeves et al., 2017).

### 5.4 Limitations and future research

This study has several limitations. First, although the study employed a three-wave time-lagged design to reduce common method bias and strengthen temporal ordering, the data were still based on self-report measures from a single source. Accordingly, the findings should not be interpreted as establishing definitive causality. Second, the sample was drawn from physicians and nurses at a single hospital in China, which may limit the generalizability of the findings to other hospitals, regions, and healthcare systems. Third, although work alienation was identified as an important partial mediating mechanism, additional processes—such as accountability tension, role ambiguity, emotional exhaustion, or technostress—may also help explain why Gen-AI-assisted clinical work collaboration contributes to instigated interprofessional incivility. Fourth, the moderation results revealed a significant but opposite-to-hypothesized role of trait EI. Although this pattern suggests that emotional resources may operate in a more context-dependent and ambivalent manner in hospital work, the present study did not directly measure contextual explanations such as collectivistic norms, relational sensitivity, conflict-avoidance tendencies, or face concerns. In addition, because trait EI is a relatively stable personality characteristic, its practical malleability through short-term interventions may be limited; the present findings therefore speak more to differential vulnerability and risk identification than to immediate personality modification.

Future research could address these limitations in several ways. First, the present study focused primarily on the demand-inducing side of Gen-AI-assisted clinical work collaboration, future research could directly compare the relative effects of resource-dominant and demand-dominant Gen-AI conditions in healthcare work. Secondly, researchers may adopt multi-source, experimental, or mixed method designs to provide a more robust test of the mechanisms proposed in this study and to strengthen causal inference. Third, future studies should replicate the model across multiple hospitals and, where possible, across different national and cultural contexts to examine the robustness and boundary conditions of the present findings. Fourth, future research could incorporate additional mediators, such as technostress, perceived devaluation of professional expertise, accountability pressure, or emotional exhaustion, to provide a more comprehensive understanding of the darker side of Gen-AI-assisted clinical work collaboration. Finally, the moderation results revealed a significant but opposite-to-hypothesized role of trait EI. Although this pattern suggests that emotional resources may operate in a more context-dependent and ambivalent manner in hospital work, the present study did not directly measure contextual explanations such as collectivistic norms, relational sensitivity, conflict-avoidance tendencies, or face concerns. In addition, because trait EI is a relatively stable personality characteristic, its practical malleability through short-term interventions may be limited; the present findings therefore speak more to differential vulnerability and risk identification than to immediate personality modification.

Finally, future studies could explore additional moderators. In addition, because the present study focused primarily on the demand-inducing side of Gen-AI-assisted clinical work collaboration, future research could directly compare the relative effects of resource-dominant and demand-dominant Gen-AI conditions in healthcare work. Such comparisons would provide a more complete JD-R-based understanding of how Gen-AI-assisted collaboration simultaneously generates motivational benefits and strain-related costs. Future research may also examine whether more malleable emotional and relational constructs, such as emotional regulation strategies, communication competence, or team emotional climate, operate alongside or instead of trait EI as boundary conditions in hospital work.

## 6 Conclusion

Drawing on human–AI collaboration research and the strain process within JD-R theory, the study tested a model in which Gen-AI-assisted clinical work collaboration influences instigated interprofessional incivility both directly and indirectly through work alienation, while trait emotional intelligence and its four dimensions condition the alienation–incivility relationship. The findings suggest that Gen-AI-assisted clinical work collaboration may heighten incivility through both immediate relational tension and a more internalized process of work alienation. In addition, the moderating effect of trait EI was significant but opposite in direction to expectations, pointing to a more context-dependent and ambivalent role of emotional intelligence in technologically mediated clinical work. Taking together, the study indicates that the relational and psychological costs of Gen-AI-assisted collaboration in healthcare are not trivial, and that effective AI implementation should not only be effective but also meaningful work engagement, professional responsibility, and interprofessional civility.

More specifically, the results showed that Gen-AI-assisted clinical work collaboration was positively associated with both instigated interprofessional incivility and work alienation, and that work alienation was positively associated with instigated interprofessional incivility. In addition, work alienation partially mediated the relationship between Gen-AI-assisted clinical work collaboration and instigated interprofessional incivility, indicating that the influence of Gen-AI-assisted clinical work collaboration on discourteous interprofessional conduct operates through both a direct relational spillover pathway and an indirect psychologically distancing pathway (Anthony et al., 2023; Hai et al., 2025; Raisch and Krakowski, 2021). These findings extend prior research on the dark side of Gen-AI by showing that, in hospital work, technology-assisted collaboration may affect not only how clinicians perform tasks, but also how they relate to colleagues across professional boundaries (Lewis, 2023).

The moderation results further suggest that the role of trait EI in technologically mediated healthcare work is more complex than originally hypothesized. Although trait EI and its four dimensions showed significant main effects, their interaction effects with work alienation on instigated interprofessional incivility were significant in the opposite direction from that predicted. Rather than buffering the interpersonal consequences of work alienation, higher levels of trait EI appeared to strengthen the alienation–incivility relationship under the present conditions. This pattern suggests that emotional resources in high-pressure clinical environments may operate in a more context-dependent and ambivalent manner than a simple protective-resource interpretation would imply (Bakker et al., 2023).

## Data Availability

The raw data supporting the conclusions of this article will be made available by the authors, without undue reservation.
