# Supplementary material for: The dark side of Gen-AI-assisted clinical work collaboration: work alienation, trait emotional intelligence, and instigated interprofessional incivility
Source: Front Psychol. 2026 Aug 20;17:1856755. doi: 10.3389/fpsyg.2026.1856755 (PMC13537999; doi:10.3389/fpsyg.2026.1856755)
Supplement: Supplementary file 1 [file Table_1.docx]

**Supplementary Table S1. Key literature, hypotheses, and empirical tests in the conceptual model.**

| **Hypothesis** | **Proposed relationship** | **Key supporting literature** | **Empirical test in manuscript** |
| --- | --- | --- | --- |
| H1 | Gen-AI-assisted clinical work collaboration → Instigated interprofessional incivility | Anthony et al. (2023); Raisch and Krakowski (2021); Bakker and Demerouti (2017); Lewis (2023) | Table 3 |
| H2 | Gen-AI-assisted clinical work collaboration → Work alienation | Anthony et al. (2023); Hai et al. (2025); Ruan et al. (2025); Bakker et al. (2023) | Table 3 |
| H3 | Work alienation → Instigated interprofessional incivility | Shantz et al. (2014); Chiaburu et al. (2014); Blau and Andersson (2005); Lewis (2023) | Table 3 |
| H4 | Gen-AI-assisted clinical work collaboration → Work alienation → Instigated interprofessional incivility | Raisch and Krakowski (2021); Shantz et al. (2014); Hai et al. (2025); Bakker et al. (2023) | Table 4 |
| H5a | Work alienation × Self-emotion appraisal → Instigated interprofessional incivility | Petrides et al. (2016); Wong and Law (2002); Ma and Liu (2019) | Table 5; Figure 2 |
| H5b | Work alienation × Others' emotion appraisal → Instigated interprofessional incivility | Petrides et al. (2016); Wong and Law (2002); Ma and Liu (2019) | Table 5; Figure 2 |
| H5c | Work alienation × Use of emotion → Instigated interprofessional incivility | Petrides et al. (2016); Wong and Law (2002); Powell et al. (2024) | Table 5; Figure 2 |
| H5d | Work alienation × Regulation of emotion → Instigated interprofessional incivility | Petrides et al. (2016); Wong and Law (2002); Powell et al. (2024) | Table 5; Figure 2 |
| H6 | Work alienation × Trait emotional intelligence → Instigated interprofessional incivility | Bakker and Demerouti (2017); Petrides et al. (2016); Wong and Law (2002); Zadorozhny et al. (2024) | Table 5; Figure 2 |
